# Chronic respiratory disease and survival outcomes after extracorporeal membrane oxygenation

**DOI:** 10.1186/s12931-021-01796-8

**Published:** 2021-07-05

**Authors:** Tak Kyu Oh, Hyoung-Won Cho, Hun-Taek Lee, In-Ae Song

**Affiliations:** 1 Department of Anesthesiology and Pain Medicine, Seoul National University Bundang Hospital, Gumi-ro, 173, Beon-gil, Bundang-gu, Seongnam, 13620 South Korea; 2 Department of Cardiology, Cardiovascular Center, Seoul National University Bundang Hospital, Gumi-ro, 173, Beon-gil, Bundang-gu, Seongnam, 13620 South Korea

**Keywords:** Acute respiratory distress syndrome, Asthma, COPD, Extracorporeal membrane oxygenation, Interstitial lung diseases, Lung cancer

## Abstract

**Background:**

Quality of life following extracorporeal membrane oxygenation (ECMO) therapy is an important health issue. We aimed to describe the characteristics of patients who developed chronic respiratory disease (CRD) following ECMO therapy, and investigate the association between newly diagnosed post-ECMO CRDs and 5-year all-cause mortality among ECMO survivors.

**Methods:**

We analyzed data from the National Health Insurance Service in South Korea. All adult patients who underwent ECMO therapy in the intensive care unit between 2006 and 2014 were included. ECMO survivors were defined as those who survived for 365 days after ECMO therapy. Chronic obstructive pulmonary disease (COPD), asthma, interstitial lung disease, lung cancer, lung disease due to external agents, obstructive sleep apnea, and lung tuberculosis were considered as CRDs.

**Results:**

A total of 3055 ECMO survivors were included, and 345 (11.3%) were newly diagnosed with CRDs 365 days after ECMO therapy. The prevalence of asthma was the highest at 6.1% (185). In the multivariate logistic regression, ECMO survivors who underwent ECMO therapy for acute respiratory distress syndrome (ARDS) or respiratory failure had a 2.00-fold increase in post-ECMO CRD (95% confidence interval [CI]: 1.39 to 2.89; *P* < 0.001). In the multivariate Cox regression, newly diagnosed post-ECMO CRD was associated with a 1.47-fold (95% CI: 1.17 to 1.86; *P* = 0.001) higher 5-year all-cause mortality.

**Conclusions:**

At 12 months after ECMO therapy, 11.3% of ECMO survivors were newly diagnosed with CRDs. Patients who underwent ECMO therapy for ARDS or respiratory failure were associated with a higher incidence of newly diagnosed post-ECMO CRD compared to those who underwent ECMO for other causes. Additionally, post-ECMO CRDs were associated with a higher 5-year all-cause mortality. Our results suggest that ECMO survivors with newly diagnosed post-ECMO CRD might be a high-risk group requiring dedicated interventions.

**Supplementary Information:**

The online version contains supplementary material available at 10.1186/s12931-021-01796-8.

## Background

Extracorporeal membrane oxygenation (ECMO) has been used in the intensive care unit (ICU) as a choice of rescue therapy to treat refractory cardiac and/or respiratory failure [1, 2]. The diseases associated with ECMO therapy include heart failure, heart inflammation, intractable arrhythmia, pulmonary hypertension, severe trauma, acute respiratory distress syndrome (ARDS), and post-cardiac surgery management [3–7]. The use of ECMO therapy has increased in the United States [8, 9], South Korea [10], and Germany [11]. Furthermore, ECMO has been widely applied as a rescue therapy in patients with ARDS or respiratory failure due to coronavirus disease-2019 (COVID-19), globally [12, 13].

Although critically ill patients requiring ECMO therapy showed a high mortality rate of 54% [14], approximately half of the ECMO patients survived until discharge. In addition, in South Korea, the hospital survival rate among critically ill patients who underwent ECMO therapy due to respiratory failure has been improving in recent years [15]. Therefore, the quality of life (QOL) in ECMO survivors has emerged as an important public health issue [16–19]. One of the most important factors that might affect the worsening QOL in ECMO survivors is the decreased pulmonary function after discharge from the hospital. A recent retrospective cohort study reported that there was a 47% reduction in the diffusion capacity of carbon monoxide and 82% of lung parenchymal pathologic changes in 38 ECMO survivors from respiratory failure [19]. Another retrospective study reported that 21 ECMO survivors from ARDS showed lung fibrosis, and minor pulmonary function abnormalities remained common at the 365-day follow-up period [17]. However, another prospective cohort study recently reported that pulmonary function was almost normal in 33 ECMO survivors from ARDS at the 365-day follow-up period [16]. Thus, the worsening QOL in ECMO survivors due to decreased pulmonary function remains controversial, and its impact on long-term mortality has not yet been clarified.

Therefore, we aimed to describe the characteristics of patients who developed CRD following ECMO therapy, and investigate the association between newly diagnosed post-ECMO CRDs and 5-year all-cause mortality among ECMO survivors. We used data regarding disease diagnosis and mortality for the entire national population of South Korea from the National Health Insurance Service (NHIS) database.

## Methods

### Study design, and ethical concerns

As a population-based cohort study, the approval of study protocol was exempted by the Institutional Review Board of the Seoul National University Bundang Hospital (approval number: X-2001-586-902) and the Health Insurance Review and Assessment Service. The requirement for informed consent was waived because the study retrospectively analyzed anonymized data.

### Data source: NHIS database in South Korea

Health records were extracted from the NHIS database for this study. In South Korea, all diagnoses of disease and prescription of drugs and/or procedures should be registered in the NHIS database for patients to receive financial coverage for any treatment charges. The diagnoses of diseases, in the NHIS database, are registered using the International Classification of Diseases 10th revision (ICD-10). Data were extracted by an independent medical record technician (S.Y. Park), who had no conflicts of interest relative to this study, at the NHIS center on April 3, 2020.

### Study population: ECMO survivors

In this study, we initially screened all adult patients (≥ 18 years old) who underwent ECMO therapy in the ICU following hospitalization between 2006 and 2014 (9 years). However, patients in whom ECMO therapy was initiated in the operating room and continued in the ICU were also included in this study. Cases of Novalung therapy were not considered as having undergone ECMO. ECMO survivors were defined as those who survived over 365 days after initiation of ECMO therapy. We were unable to classify the type of ECMO therapy such as venoarterial (VA)- or venovenous (VV)-ECMO, because NHIS database did not distinguish the prescription code of VA- and VV-ECMO.

### Exposure variable: CRD

The following diseases were considered CRDs: chronic obstructive pulmonary disease (COPD, J44), interstitial lung disease (ILD, J84.9), asthma (J45), lung cancer (C34), lung disease due to external agents (J60-J70), obstructive sleep apnea (OSA, G47.33), and lung tuberculosis (TB, A15). Their data were extracted using ICD-10 codes, and information regarding diagnostic tool such as pulmonary function test or chest computed tomography was not used for determining CRDs. Patients who were newly diagnosed within 365 days after initiation of ECMO therapy in ECMO survivors constituted the post-ECMO CRDs group. Patients who were diagnosed with CRDs within 365 days before initiation of ECMO therapy were included in a separate group. Patients who had not been diagnosed with a CRD within 365 days both before and after ECMO therapy constituted the control group. In South Korea, patients who require medication or surgical procedures for COPD, asthma, or lung cancer, should be registered in the NHIS database using ICD-10 codes by a physician to receive financial coverage for treatment.

### Study endpoint

The primary endpoint of this study was the 5-year all-cause mortality in ECMO survivors, which was defined as death within 5 years of undergoing ECMO therapy. The dates of death were extracted until December 31, 2019, and survival times were calculated from the date on which ECMO therapy was started to the date of death or December 31, 2019, whichever occurred first. The secondary endpoint of this study was the new development of post-ECMO CRDs, which was evaluated within 365 days from the date of initiation of ECMO therapy.

### Confounders

The data on confounders included demographic characteristics (age, sex, and place of residence at the time of ECMO therapy (Seoul, metropolitan city, and other areas), year of ECMO therapy, annual income level at the time of ECMO therapy, underlying disability, pre-ECMO CRD diagnoses, and Charlson comorbidity index. The Charlson comorbidity index was calculated as the sum of the 17 individual underlying diseases in Additional file 1: Table S1; thus, the Charlson comorbidity index ranged from 0 (best) to 27 (worst) in this study [20]. In South Korea, all individuals with disabilities are registered in the NHIS database to receive various benefits from the social welfare system. Disability was assigned one of six severity grades, and we considered two severity groups (1–3, severe disability; and 4–6, mild to moderate disability).

In addition, the length of hospital stays (days), duration of ECMO therapy (days), and annual volume of ECMO therapy were included as confounders. The annual volume of ECMO therapy was calculated as the total number of ECMO cases/9 years to reflect the capability of the ECMO centers, because high-volume ECMO centers are known to improve survival outcomes of ECMO patients [21]. The annual volume of ECMO therapy was divided into four groups (Q1 < 14, Q2: 15–29, Q3: 30–64, and Q4 > 64). We classified the ECMO survivors into three groups based on the main diagnosis at ECMO therapy: (1) cardiovascular group (cardiovascular disease, shock, or post-cardiac arrest), (2) respiratory group (ARDS or respiratory failure), and (3) others.

### Statistical analysis

The clinicopathological characteristics of the ECMO survivors are presented as means with standard deviations for continuous variables and numbers with percentages for categorical variables. To compare the clinicopathological characteristics between the three groups (control, pre-ECMO CRD, and post-ECMO CRD), one-way ANOVA and Chi-square tests were used for continuous variables and categorical variables, respectively. Next, we constructed a multivariate logistic regression model for the new development of post-ECMO CRDs within 365 days of ECMO therapy. All covariates were included in the multivariate logistic regression model for adjustment, except for pre-ECMO CRDs, which were not included because the pre-ECMO CRD group had been diagnosed and treated for CRD both before and after ECMO therapy. The Hosmer–Lemeshow test was used to test the goodness of fit in the multivariate logistic regression model. We also constructed a multivariate Cox regression model for 5-year all-cause mortality in survivors (model 1). All covariates were included in the multivariate model for adjustment including pre-ECMO CRD, and survival time was set as duration, and death from any cause during 5-year was set as an event in the time-to-event analysis. To examine the impact of individual post-ECMO CRDs (7 types of CRDs), another multivariate Cox regression model (model 2) was constructed to avoid multicollinearity with multivariate model 1. Finally, subgroup analyses according to the main diagnosis at ECMO therapy considering the type of ECMO were performed using multivariate Cox regression modelling. We confirmed that there was no multicollinearity in all multivariate models involving the entire cohort, with a variance inflation factor of < 2.0. The results of the Cox regression were presented as hazard ratios (HRs) with 95% confidence intervals (CIs), and log–log plots were used to confirm that the multivariate Cox model was satisfied with the Cox proportional hazard model. The results of the logistic regression models were presented as odds ratios (ORs) with 95% CIs. R software (version 4.0.3; R Foundation for Statistical Computing, Vienna, Austria) was used for all analyses, and a *P*-value < 0.05 was considered statistically significant.

## Results

### Study population

**Fig. 1:**
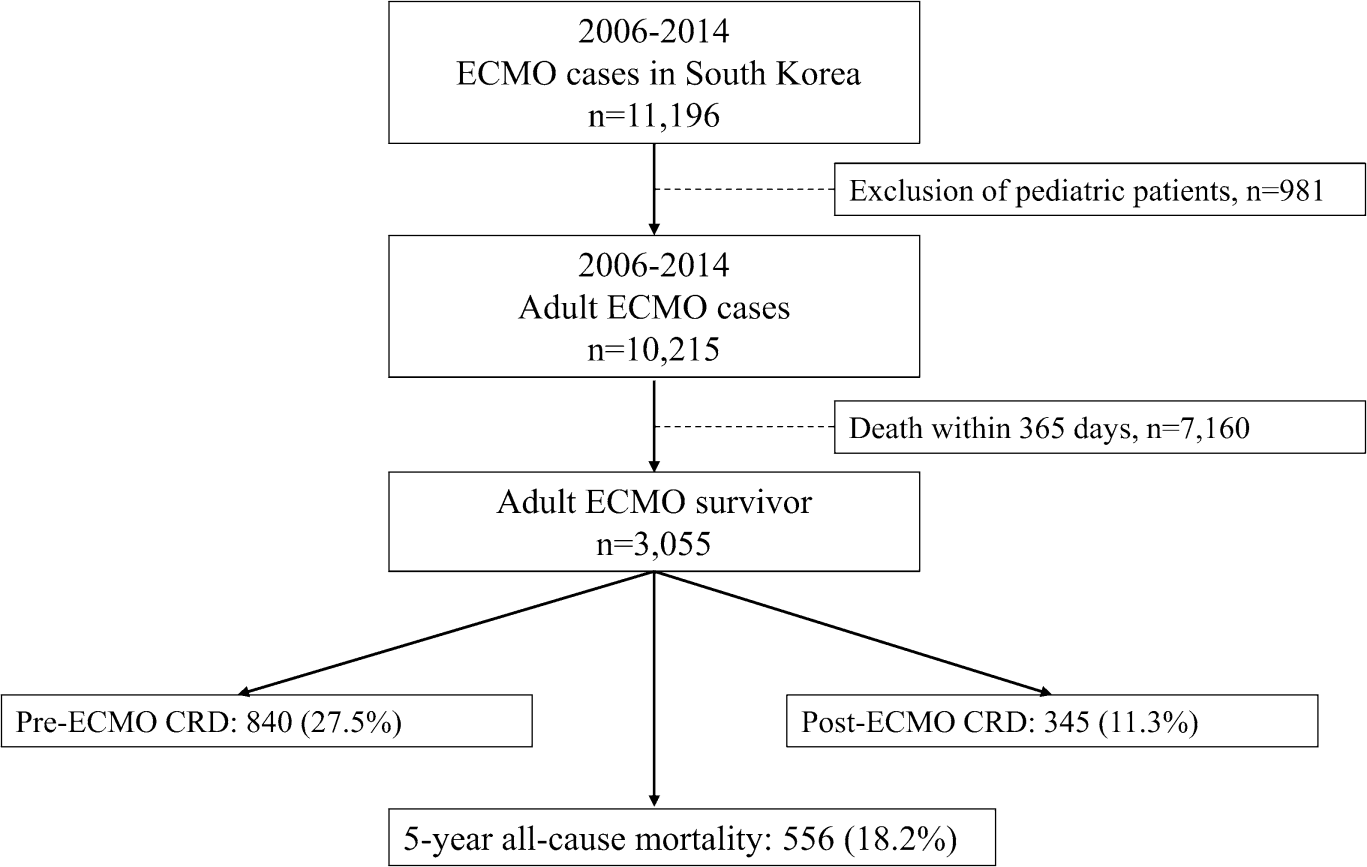
Flowchart depicting patient selection. *ECMO* extracorporeal membrane oxygenation, *CRD* chronic respiratory disease

From 2006 to 2014, 11,196 patients underwent ECMO therapy in 72 hospitals in South Korea. After excluding 981 patients who were aged < 18 years, 10,215 adult ECMO patients were screened. Of these patients, 3055 (29.9%) survived for more than 365 days after the initiation of ECMO therapy and were included in the final analysis. Among them, 840 (27.5%) and 345 (11.3%) were assigned to the pre- and post-ECMO CRD groups, respectively. Five-year all-cause mortality occurred in 556 (18.2%) ECMO survivors. Figure 1 shows a flowchart depicting patient selection. The clinicopathological characteristics of the patients are shown in Table 1. The results of the comparison of the clinicopathological characteristics between the three groups (control, pre-ECMO CRD, and post-ECMO CRD) are shown in Additional file 2: Table S2. The 5-year mortality rate in the post-ECMO CRD group was the highest (28.4%; 98), followed by the pre-ECMO CRD group (21.2%; 178), and the control group (15.0%; 280). In addition, the proportions of the respiratory group in terms of the main diagnosis associated with ECMO therapy were the highest in the post-ECMO CRD group (16.8%; 58), followed by the pre-ECMO CRD group (11.7%; 98) and the control group (6.7%; 125).

**Table 1:** The clinicopathological characteristics of the patients

Variable	Number (%)	Mean (SD)
Age, year		53.4 (15.2)
Sex, male	2032 (66.5)	
Residence at ECMO treatment		
Capital city (Seoul)	770 (25.2)	
Other metropolitan city	631 (20.7)	
Other area	1654 (54.1)	
Year of ECMO treatment		
2006	114 (3.7)	
2007	144 (4.7)	
2008	186 (6.1)	
2009	223 (7.3)	
2010	271 (8.9)	
2011	342 (11.2)	
2012	439 (14.4)	
2013	580 (19.0)	
2014	756 (24.7)	
Annual income level at ECMO treatment		
Q1 (Lowest) or unknown	748 (24.5)	
Q2	535 (17.5)	
Q3	763 (25.0)	
Q4 (Highest)	1009 (33.0)	
Annual case volume of ECMO therapy		
Q1 < 19	508 (16.6)	
Q2: 19–43	735 (24.1)	
Q3: 44–102	100 (34.4)	
Q4 > 102	762 (24.9)	
Charlson comorbidity index		2.8 (2.2)
Underlying disability		
Mild to moderate	464 (15.2)	
Severe	340 (11.1)	
Length of hospital stay, day		24.6 (15.2)
Duration of ECMO therapy, day		6.2 (10.3)
5-year all-cause mortality	556 (18.2)	
Main diagnosis at ECMO treatment		
Cardiovascular group	1930 (63.2)	
Respiratory group	281 (9.2)	
Others	844 (27.6)	

*SD* standard deviation, *ECMO* extracorporeal membrane oxygenation

### Development of post-ECMO CRD

The prevalence of pre- and post-ECMO CRDs are shown in Additional file 3: Table S3. In the pre-ECMO CRD group (840, 27.5%), the prevalence of asthma was the highest at 16.9% (516), followed by that of COPD at 8.1% (247), and lung disease due to external agents at 3.5% (106). In the post-ECMO CRD group, the prevalence of asthma was the highest at 6.1% (185), followed by that of COPD at 3.0% (91) and lung disease due to external agents at 2.2% (68). Table 2 shows the results of the multivariate logistic regression model for the development of post-ECMO CRDs among ECMO survivors. Older age (OR: 1.02, 95% CI: 1.01 to 1.03; *P* < 0.001) and longer duration of ECMO therapy (OR: 1.02, 95% CI: 1.01 to 1.03; *P* < 0.001) were associated with an increase in post-ECMO CRDs. In addition, the patients in the respiratory group who underwent ECMO therapy due to ARDS or respiratory failure had a 2.00-fold increase in post-ECMO CRD (OR: 2.00, 95% CI: 1.39 to 2.89; *P* < 0.001), compared to the patients in the cardiovascular group.

**Table 2:** Multivariable logistic regression model for the development of post-ECMO CRDs among ECMO survivors

Variable	Multivariable model	*P*-value
	OR (95% CI)	
Age, year	1.02 (1.01, 1.03)	< 0.001
Sex, male	1.00 (0.78, 1.28)	0.987
Residence at ECMO treatment		
Capital city (Seoul)	1	
Other metropolitan city	0.88 (0.62, 1.27)	0.502
Other area	1.09 (0.82, 1.46)	0.546
Year of ECMO treatment		
2006	1	
2007	2.17 (0.92, 5.15)	0.078
2008	1.26 (0.52, 3.06)	0.615
2009	1.10 (0.46, 2.67)	0.826
2010	1.22 (0.53, 2.84)	0.638
2011	1.58 (0.71, 3.53)	0.267
2012	1.28 (0.58, 2.86)	0.539
2013	1.72 (0.79, 3.72)	0.171
2014	1.81 (0.84, 3.88)	0.129
Annual income level at ECMO treatment		
Q1 (Lowest) or unknown	1	
Q2	1.29 (0.87, 1.89)	0.201
Q3	1.28 (0.90, 1.82)	0.174
Q4 (Highest)	1.88 (1.36, 2.59)	< 0.001
Annual case volume of ECMO therapy		
Q1 < 19	1	
Q2: 19–43	1.03 (0.75, 1.41)	0.859
Q3: 44–102	0.67 (0.48, 0.93)	0.017
Q4 > 102	0.54 (0.37, 0.79)	0.002
Charlson comorbidity index, point	1.05 (0.99, 1.10)	0.088
Underlying disability		
Mild to moderate	1.18 (0.84, 1.64)	0.344
Severe	1.81 (1.29, 2.53)	0.001
Duration of ECMO therapy, day	1.02 (1.01, 1.03)	< 0.001
Length of hospital day, day	1.00 (0.99, 1.01)	0.675
Main diagnosis at ECMO therapy		
Cardiovascular group	1	
Respiratory group	2.00 (1.39, 2.89)	< 0.001
Others	1.21 (0.99, 1.01)	0.675

Hosmer Lemeshow test: Chi-square, 4.08, df = 8, *P* = 0.850

*ECMO* extracorporeal membrane oxygenation, *CRD* chronic respiratory disease, *OR* odds ratio, *CI* confidence interval

### Post-ECMO CRD and five-year all-cause mortality

Table 3 shows the results of the multivariate Cox regression models for 5-year all-cause mortality among ECMO survivors. In multivariate model 1, post-ECMO CRD was associated with a 1.47-fold (HR: 1.47, 95% CI: 1.17 to 1.86; *P* = 0.001) higher 5-year all-cause mortality compared with the control group. In the multivariate model 2, post-ECMO asthma and post-ECMO ILD were associated with a 1.54-fold (HR: 1.54, 95% CI: 1.13 to 2.11; *P* = 0.007) and 2.33-fold (HR: 2.33, 95% CI: 1.05 to 6.40; *P* = 0.042) higher 5-year all-cause mortality compared with the control group, respectively. Among the covariates in multivariate model 1, pre-ECMO CRD was associated with a 1.25-fold (HR: 1.25, 95% CI: 1.04 to 1.49; *P* = 0.019) higher 5-year all-cause mortality compared with the control group. Table 4 shows the results of the subgroup analysis according to the main diagnosis at ECMO therapy. Among ECMO survivors who underwent ECMO therapy due to cardiovascular disease, shock, or post-cardiac arrest (cardiovascular group), post-ECMO CRD was associated with a 1.51-fold (HR: 1.51, 95% CI: 1.10 to 2.08; *P* = 0.011) higher 5-year all-cause mortality compared with the control group.

**Table 3:** Multivariable Cox regression models for year all-cause mortality among ECMO survivors

Variable	Multivariable model	*P*-value
	HR (95% CI)	
Multivariable model 1		
Post-ECMO CRD (vs control)	1.47 (1.17, 1.86)	0.001
Multivariable model 2		
Post-ECMO CRD		
Post-ECMO COPD	1.19 (0.80, 1.79)	0.391
Post-ECMO Asthma	1.54 (1.13, 2.11)	0.007
Post-ECMO ILD	2.33 (1.05, 6.40)	0.042
Post-ECMO Lung cancer	2.21 (0.81, 6.03)	0.123
Post-ECMO Lung disease due to external agent	1.07 (0.65, 1.77)	0.787
Post-ECMO OSA	3.60 (0.48, 27.08)	0.214
Post-ECMO TB	1.29 (0.67, 2.49)	0.443
Other variables in model 1		
Age, year	1.04 (1.04, 1.05)	< 0.001
Sex, male (vs female)	1.18 (0.98, 1.41)	0.087
Residence at ECMO treatment		
Capital city (Seoul)	1	
Other metropolitan city	0.85 (0.65, 1.12)	0.250
Other area	0.95 (0.77, 1.17)	0.633
Year of ECMO treatment		
2006	1	
2007	0.70 (0.41, 1.18)	0.176
2008	0.50 (0.30, 0.86)	0.012
2009	0.55 (0.33, 0.92)	0.022
2010	0.82 (0.52, 1.30)	0.406
2011	0.78 (0.50, 1.21)	0.266
2012	0.78 (0.51, 1.20)	0.264
2013	0.56 (0.37, 0.87)	0.009
2014	0.63 (0.42, 0.96)	0.030
Annual income level at ECMO treatment		
Q1 (Lowest)	1	
Q2	0.94 (0.72, 1.21)	0.615
Q3	0.67 (0.52, 0.86)	0.001
Q4 (Highest)	0.84 (0.67, 1.056)	0.120
Annual case volume of ECMO therapy		
Q1 < 19	1	
Q2: 19–43 Q3: 44–102	0.76 (0.58, 0.99)	0.039
	1.11 (0.88., 1.41)	0.379
Q4 > 102	0.91 (0.69, 1.21)	0.525
Charlson comorbidity index, point	1.05 (1.02, 1.09)	0.006
Pre-ECMO CRD (vs control)	1.25 (1.04, 1.49)	0.019
Underlying disability		
Mild to moderate	0.86 (0.67, 1.11)	0.251
Severe	1.92 (1.53, 2.41)	< 0.001
Length of hospital stay, day	1.00 (0.99, 1.01)	0.795
Duration of ECMO therapy, day	1.03 (1.02, 1.03)	< 0.001
Main diagnosis at ECMO treatment		
Cardiovascular group	1	
Respiratory group	1.34 (0.97, 1.85)	0.076
Others	2.19 (1.78, 2.68)	< 0.001

*ECMO* extracorporeal membrane oxygenation, *HR* hazard ratio, *CI* confidence interval, *CRD* chronic respiratory disease, *COPD* chronic obstructive pulmonary disease, *ILD* interstitial lung disease, *OSA* osbsructive sleep apnea, *TB* tuberculosis of lung

**Table 4:** Subgroup analysis according to the main diagnosis at ECMO therapy, depending on the type of ECMO

Variable	Multivariable model	*P*-value
	HR (95% CI)	
Cardiovascular group		
Control	1	
Post-ECMO CRD	1.51 (1.10, 2.08)	0.011
Respiratory group		
Control	1	
Post-ECMO CRD	1.74 (0.77, 3.94)	0.185
Others		
Control	1	
Post-ECMO CRD	1.28 (0.94, 1.74)	0.112

*ECMO* extracorporeal membrane oxygenation, *HR* hazard ratio, *CI* confidence interval, *CRD* chronic respiratory disease

## Discussion

This population-based cohort study showed that 11.3% of patients were newly diagnosed with CRDs within 365 days after ECMO therapy, and asthma and COPD were most common in the post-ECMO CRDs. Older age, severe underlying disability, longer duration of ECMO therapy, and ECMO therapy due to ARDS or respiratory failure were associated with higher incidence of post-ECMO CRDs. Additionally, post-ECMO CRDs were associated with higher 5-year all-cause mortality among ECMO survivors. Our results suggest that QOL might worsen due to the development of CRDs after ECMO therapy, worsening long-term survival. We believe that our results are important because the number of ECMO survivors is bound to increase in the future due to the COVID-19 pandemic [22], and the QOL in ECMO survivors can be an important public health issue.

The results of our study should be interpretated carefully as we were unable to demonstrate that ECMO therapy was independently associated with post-ECMO CRD. It is possible that underlying the diseases that required ECMO therapy could have led to the development of post-ECMO CRDs among survivors. The Charlson comorbidity index among patients in the post-ECMO CRD group was significantly higher than that in the control group (Additional file 2: Table S2), suggesting that the patients with post-ECMO CRD were at higher risk of developing CRD after ECMO therapy than those in the control group. In addition, although the longer duration of ECMO therapy was associated with a higher incidence of post-ECMO CRD, the OR was very low (1.02; 95% CI: 1.01, 1.03); hence, its clinical significance might be limited.

Our findings are notable in that we focused on the impact of newly diagnosed CRD among the patients in the cardiovascular group who had a higher possibility of VA-ECMO than VV-ECMO therapy. VA-ECMO therapy is known to cause lung injury through multiple mechanisms [23]. First, the initiation of VA-ECMO triggers more serious systemic inflammatory response syndrome (SIRS) than that due to VV-ECMO, and SIRS-related acute lung injury may occur in patients undergoing VA-ECMO [24]. Second, the left ventricular (LV) pressure and afterload can be increased during VA-ECMO therapy, which can result in pulmonary congestion and edema [25]. VA-ECMO-induced pulmonary congestion and edema may lead to alveolar hypoxia, which causes and maintain SIRS through a vicious cycle [26]. Third, the pulmonary capillary blood flow and blood flow through the bronchial arteries were decreased during VA-ECMO therapy, which may result in lung ischemia [27, 28]. Ultimately, these lung injuries during VA-ECMO therapy can cause persisting lung inflammation and fibrosis, and could result in long-term lung dysfunction among survivors of VA-ECMO [23]. In our study, 195/1930 (10.1%) patients in the cardiovascular group were newly diagnosed with CRD within 365-days following ECMO therapy, and it significantly worsened long-term survival in the subgroup analysis (Table 4). Our results are valuable since we report that post-ECMO CRD might also be clinically important among the cardiovascular group. However, the etiology of CRD after VA-ECMO therapy remains controversial, and further studies, in this regard, are warranted.

The findings in our study are based on the diagnostic codes registered by ICD-10 in the NHIS database, and they differed from previous studies [16, 17, 19], which evaluated decreased pulmonary function using diagnostic tests such as computed tomography. Our approach has both merits and disadvantages in evaluating pulmonary function among ECMO survivors. As a merit, we enrolled a relatively large sample size of 3,055 ECMO survivors and followed up their 5-year mortality using a national database. However, as a disadvantage, our study could not include some cases of CRDs, because some patients with CRDs might not visit outpatient clinics for various reasons such as low compliance to treatment, economic burden, and poor accessibility to medical resources. To interpret our results in the context of those presented in other studies [16, 17, 19], these differences in method should be considered.

In this study, respiratory group in terms of main diagnosis at ECMO therapy was associated with the development of post-ECMO CRDs. The respiratory group had a twofold higher odds of post-ECMO CRD development than those in the cardiovascular group. Therefore, our results suggest that pulmonary sequelae were developed in ECMO survivors without pre-ECMO CRDs after ECMO therapy due to ARDS or respiratory failure, as previously reported [17, 19].

A longer duration of ECMO therapy was also associated with a higher incidence of post-ECMO CRDs among survivors, suggesting there might be a dose-dependent effect. Longer duration of ECMO therapy means that the survivors failed early weaning of ECMO. The longer duration of ECMO therapy has been known to be a risk factor for higher mortality in patients undergoing both VA- and VV-ECMO therapy [15, 29, 30]. For survivors of ECMO, a recent retrospective cohort study showed that a longer duration of ECMO therapy was associated with a lower rate of complete recovery [31]. Another study reported that the usual duration of ECMO in patients with ARDS was 7–10 days, and patients with prolonged duration ECMO (> 14 days) were at a higher risk of mortality with irreversible lung injury [29]. In this study, we showed that the prolonged duration of ECMO therapy was a significant risk factor for newly diagnosed CRDs among survivors.

Interestingly, asthma and COPD were the most common among all post-ECMO CRDs. The accurate mechanism of ECMO therapy with new diagnoses of asthma or COPD has not been identified yet. One possible mechanism is the impact of systemic inflammation during ECMO therapy. Lung and systemic inflammation have been known as key factors in the pathogenesis of asthma and COPD [32]. Exposure to the ECMO circuit causes both systemic and lung inflammatory reactions [33]. Thus, the inflammatory reaction might contribute to the new development of asthma and COPD in the post-ECMO period among survivors. However, the information regarding this phenomenon is lacking, and more research is needed to confirm the relationship between ECMO therapy and development of asthma or COPD.

Our study had several limitations. First, some important variables, including body mass index, alcohol abuse, and smoking history, were not included in the analysis because the necessary information was not available in the NHIS database. Second, we defined comorbidities using their ICD-10 codes to calculate the Charlson comorbidity index; however, the ICD-10 codes might not accurately reflect the actual diseases in the study population. Third, we did not distinguish between VA- and VV-ECMO in this study because of the limited prescription codes for ECMO in South Korea. Therefore, information on the difference in the prevalence of CRD between VA- and VV-ECMO was missing in our study. However, we classified the ECMO survivors considering the main diagnosis at ECMO therapy into the cardiovascular group, respiratory group, and other group. In a recent cohort study, patients who underwent ECMO therapy with a primary diagnosis of acute respiratory failure constituted the VV-ECMO group, while patients who underwent ECMO therapy with a primary diagnosis of cardiogenic shock or cardiac arrest constituted the VA-ECMO group [34]. Thus, our classification using main diagnosis at ECMO therapy might provide some important information for readers on the effect of type of ECMO. Fourth, as mentioned above, the diagnosis of CRD was possibly underestimated because not all patients with CRDs were assessed properly. For example, some patients with CRDs might not visit the outpatient clinic for the treatment of CRDs due to mild symptoms or limited access to medical resource utilization, and they could not be included in this study. Fifth, we did not use diagnostic tools such as pulmonary function test or chest computed tomography for determining post-ECMO CRDs. Lastly, we did not assess the cause of 5-year mortality among survivors of ECMO due to the limitations of our data source.

## Conclusions

In conclusion, at 12 months after ECMO therapy, 11.3% of ECMO survivors were newly diagnosed with CRDs, and asthma and COPD were the most common. Factors such as old age, disability, longer duration of ECMO therapy, and ECMO therapy due to ARDS or respiratory failure were associated with the development of post-ECMO CRDs. Additionally, post-ECMO CRDs were associated with higher 5-year all-cause mortality among ECMO survivors.

## Supplementary Information

**Additional file 1: Table S1.** ICD-10 codes used to compute the Charlson comorbidity index.

**Additional file 2: Table S2.** Results of the comparison of the clinicopathological characteristics between the three groups.

**Additional file 3: Table S3.** Prevalence of pre- and post-ECMO CRDs.

## Data Availability

All data will be available upon reasonable request to the corresponding author.

## Abbreviations

ARDS: Acute respiratory distress syndrome
CI: Confidence interval
COPD: Chronic obstructive pulmonary disease
COVID-19: Coronavirus disease-2019
CRD: Chronic respiratory disease
ECMO: Extracorporeal membrane oxygenation
HR: Hazard ratio
ICD-10: International Classification of Diseases 10th revision
ICU: Intensive care unit
ILD: Interstitial lung disease
NHIS: National Health Insurance Service
OR: Odds ratio
OSA: Obstructive sleep apnea
SIRS: Systemic inflammatory response syndrome
QOL: Quality of life
VA: Venoarterial
VV: Venovenous
