# Supplementary material for: Chronic respiratory disease and survival outcomes after extracorporeal membrane oxygenation
Source: Respir Res. 2021 Jul 5;22:195. doi: 10.1186/s12931-021-01796-8 (PMC8256197; doi:10.1186/s12931-021-01796-8)
Supplement: Supplementary file 2 — Additional file 2: Table S2. Results of the comparison of the clinicopathological characteristics between the three groups. [file 12931_2021_1796_MOESM2_ESM.docx]

Table S2. The results of the comparison of the clinicopathological characteristics between the three groups

| Variable | | Control | Pre-ECMO CRD | Post-ECMO CRD | *P*-value |
| --- | --- | --- | --- | --- | --- |
|  |  | n=1,870 | n=840 | n=345 |  |
| Age, year | | 52.3 (14.9) | 54.8 (15.5) | 55.9 (15.3) | <0.001 |
| Sex, male | | 1,239 (66.3) | 559 (66.5) | 234 (67.8) | 0.851 |
| Residence at ECMO treatment | |  |  |  | 0.096 |
|  | Capital city (Seoul) | 500 (26.7) | 188 (22.4) | 82 (23.8) |  |
|  | Other metropolitan city | 384 (20.5) | 183 (21.8) | 64 (18.6) |  |
|  | Other area | 986 (52.7) | 469 (55.8) | 199 (57.7) |  |
| Year of ECMO treatment | |  |  |  | 0.124 |
|  | 2006 | 70 (3.7) | 36 (4.3) | 8 (2.3) |  |
|  | 2007 | 81 (4.3) | 42 (5.0) | 21 (6.1) |  |
|  | 2008 | 109 (5.8) | 61 (7.3) | 16 (4.6) |  |
|  | 2009 | 142 (7.6) | 64 (7.6) | 17 (4.9) |  |
|  | 2010 | 174 (9.3) | 73 (8.7) | 24 (7.0) |  |
|  | 2011 | 205 (11.0) | 99 (11.8) | 38 (11.0) |  |
|  | 2012 | 267 (14.3) | 128 (15.2) | 44 (12.8) |  |
|  | 2013 | 352 (18.8) | 155 (18.5) | 73 (21.2) |  |
|  | 2014 | 470 (25.1) | 182 (21.7) | 104 (30.1) |  |
| Annual income level at ECMO treatment | |  |  |  | 0.001 |
|  | Q1 (Lowest) or unknown | 489 (26.1) | 195 (23.2) | 64 (18.6) |  |
|  | Q2 | 312 (16.7) | 166 (19.8) | 57 (16.5) |  |
|  | Q3 | 480 (25.7) | 204 (24.3) | 79 (22.9) |  |
|  | Q4 (Highest) | 589 (31.5) | 275 (32.7) | 145 (42.0) |  |
| Annual case volume of ECMO therapy | |  |  |  | <0.001 |
|  | Q1 <19 | 426 (22.8) | 200 (23.8) | 92 (26.7) |  |
|  | Q2: 19-43 | 496 (26.5) | 253 (30.1) | 111 (32.2) |  |
|  | Q3: 44-102 | 526 (28.1) | 265 (31.5) | 89 (25.8) |  |
|  | Q4 > 102 | 422 (22.6) | 122 (14.5) | 53 (15.4) |  |
| Charlson comorbidity index | | 2.5 (2.0) | 3.6 (2.5) | 2.9 (2.2) | <0.001 |
| Underlying disability | |  |  |  | <0.001 |
|  | Mild to moderate | 311 (16.6) | 99 (11.8) | 54 (15.7) |  |
|  | Severe | 221 (11.8) | 64 (7.6) | 55 (15.9) |  |
| Length of hospital stay, day | | 24.0 (15.2) | 25.4 (15.2) | 25.4 (15.3) | 0.064 |
| Treatment of ECMO, day | | 5.8 (9.4) | 5.9 (9.3) | 9.1 (15.7) | <0.001 |
| 5-year all-cause mortality | | 280 (15.0) | 178 (21.2) | 98 (28.4) | <0.001 |
| Main diagnosis at ECMO therapy | |  |  |  | <0.001 |
|  | Cardiovascular group | 1,220 (65.2) | 515 (61.3) | 195 (56.5) |  |
|  | Respiratory group | 125 (6.7) | 98 (11.7) | 58 (16.8) |  |
|  | Others | 525 (28.1) | 227 (27.0) | 92 (26.7) |  |

Presented as number with percentage or mean with standard deviation

ECMO, extracorporeal membrane oxygenation; CRD, chronic respiratory disease
