# Supplementary material for: Chronic respiratory disease and survival outcomes after extracorporeal membrane oxygenation
Source: Respir Res. 2021 Jul 5;22:195. doi: 10.1186/s12931-021-01796-8 (PMC8256197; doi:10.1186/s12931-021-01796-8)
Supplement: Supplementary file 3 — Additional file 3: Table S3. Prevalence of pre- and post-ECMO CRDs. [file 12931_2021_1796_MOESM3_ESM.docx]

Table S3. The prevalence of pre- and post-ECMO CRDs

| CRD | | Pre-ECMO CRD | Post-ECMO CRD |
| --- | --- | --- | --- |
| Total CRD | | 840 (27.5) | 345 (11.3) |
|  | COPD | 247 (8.1) | 91 (3.0) |
|  | Asthma | 516 (16.9) | 185 (6.1) |
|  | ILD | 26 (0.9) | 11 (0.4) |
|  | Lung cancer | 87 (2.8) | 11 (0.4) |
|  | Lung disease due to external agent | 106 (3.5) | 68 (2.2) |
|  | OSA | 5 (0.2) | 4 (0.1) |
|  | TB | 53 (1.7) | 32 (1.0) |

Presented as number with percentage

ECMO, extracorporeal membrane oxygenation; CRD, chronic respiratory disease; COPD, chronic obstructive pulmonary disease; ILD, interstitial lung disease; OSA, osbsructive sleep apnea; TB, tuberculosis of lung
